# Effect of probiotic supplementation on total lactobacilli, bifidobacteria and short chain fatty acids in 2–5-year-old children

**DOI:** 10.1080/16512235.2017.1298340

**Published:** 2017-03-10

**Authors:** R. Hemalatha, A. C. Ouwehand, M. T. Saarinen, U. V. Prasad, K. Swetha, V. Bhaskar

**Affiliations:** ^a^ Microbiology and Immunology Division, National Institute of Nutrition, Hyderabad, India; ^b^ Active Nutrition, DuPont Nutrition and Health, Kantvik, Finland

**Keywords:** *Lactobacillus*, *Bifidobacterium*, diarrhoea, microbial metabolites

## Abstract

​**Background**: Consumption of *Lactobacillus paracasei* Lpc-37 or *Bifidobacterium lactis* HN019 by 2–5-year-old children was found to reduce risk for diarrhoea and fever during the rainy season.

**Objective**: Can changes in faecal short chain fatty acids (SCFAs) or branched chain fatty acids (BCFAs) explain the observed positive influence of probiotics and their role on nutritional status and diarrhoea risk?

**Design**: Faecal samples were analysed for SCFAs and BCFAs and correlated to *Bifidobacterium* and *Lactobacillus* levels; both at the start and after nine months’ consumption of either of the two probiotic strains, or placebo.

**Results**: No differences in SCFAs, BCFAs, *Lactobacillus* or *Bifidobacterium* levels were found between boys and girls. Severely underweight children were observed to have the highest *Lactobacillus* levels. Probiotic intervention was found to be associated with higher levels of selected SCFAs and BCFAs in subjects who had experienced diarrhoea. Treatment with either of the probiotics led to changes in SCFAs and BCFAs. SCFAs, acetate, propionate and butyrate, were found to correlate with each other. Likewise, BCFAs isobutyrate, 2-methylbutyrate and isovalerate correlated with each other. After the intervention, *L. paracasei* Lpc-37 correlated positively with total *Bifidobacterium* counts and isovalerate levels. *B. lactis* HN019 counts were found to correlate positively with total bacterial counts and negatively with propionate levels.

**Conclusions**: ​Nutritional status was associated with higher levels of faecal lactobacilli; the meaning of this requires further investigation. The intervention with the two probiotics was observed to influence the levels of faecal SCFAs and BCFAs and there is a differential response in those who developed diarrhoea and those who did not. It is, however, not clear to what extent this is a mechanism that explains the earlier observed effect the strains had on diarrhoea risk.

## Introduction

The composition and activity of the intestinal microbiota influences intestinal and thereby faecal short chain fatty acid (SCFA) and branched chain fatty acid (BCFA) profiles. SCFAs and BCFAs have an important influence on intestinal health and are related to various health conditions. Acetic acid mainly serves as energy source for skeletal muscles, propionic acid is utilised by hepatocytes in gluconeogenesis and butyric acid is of major importance to colonic health as it is one of the main energy sources for colonocytes and is thought to be associated with a reduction in risk for various colonic diseases.[1] Besides functioning as energy sources, SCFAs have been linked to reduced risk for metabolic syndrome,[2] stimulate intestinal motility,[3] reduce serum LDL cholesterol [4] and contribute to satiety.[5]

A better understanding of the physiological levels of faecal SCFAs in children would be useful to better comprehend their role in health and pathological conditions. While much attention has been paid to the microbiota composition and activity of infants,[6,7] much less work has been done on this topic in slightly older children. Furthermore, the microbial activity, such as faecal SCFAs and BCFAs, has been studied much less in children from disadvantaged backgrounds. In the current study, we selected faecal samples of 140 children from a larger cohort (*n* = 379) of healthy children that had been enrolled in a probiotic intervention study in India.[8] The study investigated the influence of *Bifidobacterium animalis* ssp. *lactis* HN019, *Lactobacillus paracasei* Lpc-37 or placebo on the incidence of community acquired diarrhoea in 2–5-year-old, apparently healthy, children. The study indicated that both probiotic strains could reduce the incidence for community acquired diarrhoea and fever during the rainy season. However, outside the rainy season when diarrhoea and fever incidence was low, the strains had no influence on these symptoms. The study also observed that the genus *Lactobacillus* was the main member of the faecal microbiota.[8] A subset of samples of 140 children was selected such that all children that had developed diarrhoea during the study were included as well as 25 children from each intervention group that did not develop diarrhoea. From the faecal samples selected this way, we have quantified SCFAs and BCFAs. Combined with the faecal total bacteria, genus *Lactobacillus* and *Bifidobacterium* levels as reported earlier,[8] the SCFA and BCFA levels were compared by gender, nutritional status and for those children developing diarrhoea or not. Furthermore, all variables were correlated to each other at their baseline levels to determine if bifidobacteria or lactobacilli can be expected to influence the levels of the main faecal microbial metabolites.

## Methods and materials

### Volunteers and study set-up

The study set-up and volunteers recruited have been described earlier.[8] In short, the study had a one-month baseline period, a nine-month intervention period and a three-month washout period and ran between July 2010 and July 2011. Faecal samples were collected at baseline, end of intervention and at the end of the wash-out period. From Secunderabad city in South India, 379 apparently healthy children aged 2–5 years were recruited and randomised over three treatments. During the intervention period the children received either placebo (microcrystalline cellulose), *Bifidobacterium animalis* ssp. *lactis* HN019 (AGAL NM97/09513; 5 × 10^9^ CFU/day) or *Lactobacillus paracasei* Lpc-37 (ATCC SD5275; 2 × 10^9^ CFU/day). Viability of the strains was determined at the start and the end of the study; no significant loss of viability was observed during the refrigerated storage. The study products were provided as a capsule that was opened by the caretakers and mixed with 50 ml milk. The study products were manufactured, randomised and blinded by Danisco USA (Madison, WI, USA) and were indistinguishable from each other in taste, smell, colour, weight, or packaging.

Weight and height were determined from all children at baseline. Weight was measured to the nearest 100 g using digital weighing scale (SECA, Hamburg, Germany) and height was measured to the nearest centimetre using measuring height rod (GPM anthropological instruments, Zurich, Switzerland). Health status (diarrhoea) was assessed during the whole study period.

The study was approved by the Scientific Advisory Committee (SAC) as well as the Institutional Review Board (IRB) of the National Institute of Nutrition (NIN, Hyderabad, India). The study has been registered in Clinical Trial Registry India; CTRI/2012/08/002942. Written informed consent was obtained from the parents or legal guardians of all the participating children. All clinical investigations were conducted according to the principles expressed in the Declaration of Helsinki.[9]

### Faecal analyses

**Figure 1.**
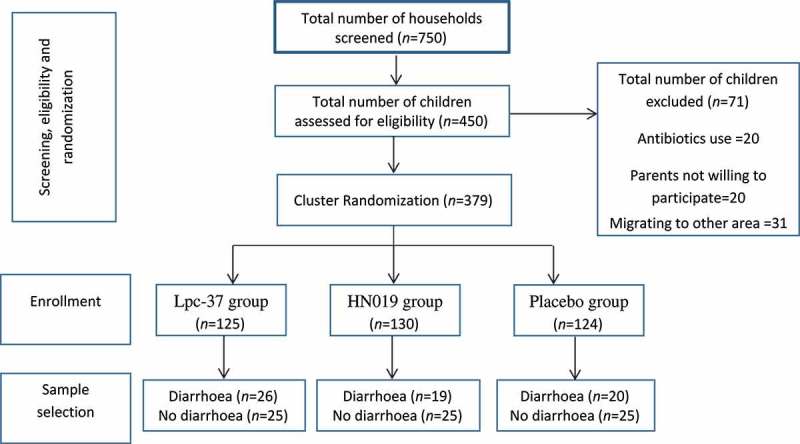
Flow diagram of the sample selection from the earlier published trial.[8]​

Faecal samples were collected at the end of each period; i.e. after baseline, intervention and washout respectively. Of the 379 children, samples from all children with diarrhoea were analysed, as well as from 25 randomly chosen children in each treatment group; in all, 140 children (Figure 1). Total faecal bacterial counts were determined by flow cytometry,[10] *Lactobacillus* and *Bifidobacterium* counts were determined by quantitative real-time PCR as reported earlier.[8] In short; primers specific for *Lactobacillus* spp.,[11] *Bifidobacterium* spp.,[12] as well as the administered probiotics, *Bifidobacterium lactis* [13] and *Lactobacillus paracasei*.[14] To obtain standard curves, a 10-fold dilution series ranging from 10 pg to 10 ng of DNA from the bacterial standard cultures (*L. paracasei* Lpc-37 and *B. lactis* HN019) were included. Analysis of short chain fatty acids (SCFAs) was performed essentially as described in [15] using gas chromatography, analysing the concentration of acetic, propionic, butyric, isobutyric, valeric, isovaleric and 2-methylbutyric.

### Statistical analysis

For the descriptive data, an independent sample *t*-test was performed. For non-normal (Gaussian distribution) data the Mann–Whitney test was used to assess significance. Normality was determined by calculating the skewness of the data for a particular variable. Values were considered significant at *p* < 0.05 with 95% CI. Spearman rank correlation was performed to study the relation between all variables such as faecal bacteria and short chain fatty acids. r (rho) values indicate correlations and p values indicate significance at 95% CI. A correction for false discoveries was made according to Bonferroni.

## Results

No statistically significant difference was observed between male and female children in levels of faecal SCFAs, Table S1. Similarly, no difference was observed in faecal SCFA of well-nourished and undernourished children (Table S2).

When comparing diarrhoea and non-diarrhoea cases, the *L. paracasei* Lpc-37 group was found to have higher levels of faecal iso-butyric and iso-valeric acid post-intervention in children with diarrhoea (Table 1). From baseline to post-intervention, there was no change over time comparing diarrhoea vs. non-diarrhoea in the *L. paracasei* Lpc-37 group. However, from post-intervention to washout there were smaller increases for children who experienced diarrhoea compared to non-diarrhoea for iso-butyric acid (0.236 vs. 1.753 respectively, *p* = 0.036), 2-methylbutyric acid (0.041 vs. 1.064 respectively, *p* = 0. 040) and iso-valeric acid (0.044 vs. 1.827 respectively, *p* = 0.016).

**Table 1.** Comparison of diarrhoea and non-diarrhoea cases by treatment (*L. paracase*i Lpc-37, *B. lacti*s HN019 and placebo) and study period (baseline, post-intervention and washout) in children (2–5 years).

		*L. paracasei* Lpc-37		*B. lactis* HN019		Placebo	
	µmol g^–1^	Non-diarrhoea (*n* = 25)	Diarrhoea (*n* = 26)	Non-diarrhoea (*n* = 25)	Diarrhoea (*n* = 19)	Non-diarrhoea (*n* = 25)	Diarrhoea (*n* = 20)
Baseline	Acetic acid	72.38 ± 29.66	73.40 ± 24.90	76.05 ± 25.35^††^	64.87 ± 15.95^††^	72.73 ± 20.82^†^	68.67 ± 24.06^†^
	Propionic acid	37.26 ± 20.21	36.95 ± 15.73	40.15 ± 21.13^††^	32.93 ± 11.89^††^	33.99 ± 18.58	37.05 ± 19.18
	Butyric acid	22.71 ± 14.93	21.97 ± 11.95	21.69 ± 9.81	20.49 ± 13.31	23.13 ± 13.20	23.98 ± 12.97
	Valeric acid	3.24 ± 3.69	3.76 ± 3.17	3.57 ± 3.55	3.13 ± 3.23	2.81 ± 3.90	3.95 ± 5.48
	Isobutyric acid	1.21 ± 1.99	1.30 ± 1.51	1.61 ± 1.50	0.99 ± 0.99	1.41 ± 1.36	0.91 ± 1.01
	2-methylbutyric acid	1.00 ± 1.41	0.75 ± 0.97	0.95 ± 1.04	0.59 ± 0.51	0.78 ± 0.82	0.52 ± 0.57
	Isovaleric acid	1.26 ± 1.78	1.40 ± 1.32	1.55 ± 1.38	1.03 ± 0.93	1.43 ± 1.17	1.13 ± 1.06
	Lactic acid	3.80 ± 5.90	7.07 ± 8.80	14.30 ± 33.00	21.62 ± 36.46	10.40 ± 23.81	18.44 ± 23.96
Post-intervention	Acetic acid	80.94 ± 26.44	83.72 ± 24.44	77.15 ± 23.87*††	91.99 ± 26.55*††	74.44 ± 20.57^†^	82.40 ± 24.57^†^
	Propionic acid	35.58 ± 20.19	40.86 ± 19.35	35.27 ± 15.11*††	44.41 ± 20.38*††	34.37 ± 15.40	36.92 ± 13.80
	Butyric acid	24.46 ± 16.70	29.81 ± 13.67	26.36 ± 12.59	28.46 ± 13.93	26.73 ± 11.16	25.35 ± 11.08
	Valeric acid	2.86 ± 2.96	4.09 ± 2.44	2.70 ± 2.94	3.70 ± 3.01	2.83 ± 1.77	3.17 ± 2.48
	Isobutyric acid	1.43 ± 1.32**††	2.22 ± 1.34**††	1.63 ± 1.60	1.41 ± 1.19	1.66 ± 1.39	1.82 ± 1.70
	2-methylbutyric acid	0.86 ± 0.87^††^	1.25 ± 1.06^††^	0.93 ± 1.11	0.64 ± 0.56	0.95 ± 0.95	1.03 ± 1.14
	Isovaleric acid	1.20 ± 1.02**††	2.25 ± 1.38**††	1.49 ± 1.48	1.59 ± 1.26	1.58 ± 1.03	1.92 ± 1.64
	Lactic acid	14.52 ± 39.44	3.08 ± 2.00	3.90 ± 10.01	12.80 ± 34.51	3.07 ± 4.20	5.15 ± 5.48
Washout	Acetic acid	100.38 ± 32.65	92.69 ± 27.42	103.88 ± 30.01^††^	91.14 ± 30.37^††^	103.13 ± 30.65^†^	95.70 ± 25.51^†^
	Propionic acid	50.82 ± 23.88	51.15 ± 26.74	55.25 ± 26.00*††	42.53 ± 17.69*††	48.80 ± 17.75	45.70 ± 19.98
	Butyric acid	42.07 ± 23.18	36.71 ± 19.62	38.52 ± 16.72	36.04 ± 17.24	35.61 ± 13.27	34.75 ± 16.90
	Valeric acid	6.23 ± 7.42	5.95 ± 6.08	5.17 ± 4.12	5.33 ± 6.28	5.67 ± 5.90	4.68 ± 3.72
	Isobutyric acid	3.19 ± 2.59^††^	2.52 ± 1.67^††^	2.78 ± 2.67	2.20 ± 1.85	3.26 ± 2.17	2.45 ± 3.01
	2-methylbutyric acid	1.93 ± 1.69^††^	1.35 ± 1.11^††^	1.69 ± 1.90	1.18 ± 1.24	2.11 ± 1.37	1.34 ± 1.89
	Isovaleric acid	3.03 ± 2.45^††^	2.37 ± 1.54^††^	2.54 ± 2.32	2.09 ± 1.56	2.90 ± 1.95	2.40 ± 2.64
	Lactic acid	7.57 ± 21.18	3.83 ± 3.32	3.36 ± 4.60	8.90 ± 27.01	4.24 ± 9.89	6.20 ± 9.23

*<0.10 comparison between diarrhoea and non-diarrhoea

**p < 0.05 comparison between diarrhoea and non-diarrhoea

^†^<0.10 comparison between following time points (i.e. baseline vs. post-intervention or post-intervention vs. washout)

^††^ *p* < 0.05 comparison between following time points (i.e. baseline vs. post-intervention or post-intervention vs. washout)

In the *B. lactis* HN019 group, post-intervention, there were trends for higher levels of faecal acetic acid and propionic acid in children with diarrhoea, while after washout, faecal propionic acid levels were higher in the group of children that had not suffered from non-diarrhoea (Table 1). From baseline to post-intervention, there were bigger increases for children who experienced diarrhoea compared to non-diarrhoea for acetic acid (27.123 vs. 1.104 respectively, *p* = 0.011) and propionic acid (11.477 vs. −4.872 respectively, *p* = 0.036). While from post-intervention to washout there were smaller increases for children who experienced diarrhoea compared to non-diarrhoea for acetic acid (−0.251 vs. 26.726 respectively, *p* = 0.029) and propionic acid (−2.854 vs. 19.979 respectively, *p* = 0.008).

In the placebo group, no differences were observed between children that had suffered from diarrhoea and those that had not (Table 1). From baseline to post-intervention, comparing children who experienced diarrhoea vs. those who did not; there was a trend for a larger increase in faecal acetic acid (18.569 vs. 1.711 *p* = 0.078), while from post-intervention to washout, there was a trend for smaller increase in acetic acid in children who experienced diarrhoea vs. those who did not (−0.251 vs. 28.692 respectively, *p* = 0.082).

At baseline, total faecal bacterial counts were 9.1–11.10 Log_10_ g^–1^ and total lactobacilli counts were 6.32–11.13 Log_10_ g^–1^, while the range of total bifidobacteria was 5.05–10.02 Log_10_ g^–1^ (Table S1). Bacterial counts were not different between boys and girls (Table S1). At baseline, total faecal bacteria, bifidobacteria and SCFAs were similar in well-nourished and undernourished children, but total faecal *Lactobacillus* counts were significantly higher in severely undernourished children compared to well-nourished and moderately undernourished children (10.57 vs. 9.79 and 9.93, respectively), Table S2.

At baseline, both faecal *Lactobacillus* and *Bifidobacterium* levels were found to be associated with each other and bifidobacteria with total faecal bacteria (Table 2). Total faecal bacterial counts were positively correlated with selected measured SCFAs and all measured BCFAs (Table 2). *Lactobacillus* was positively correlated with isobutyric and valeric acids, but not with the other measured SCFAs and BCFAs. *Bifidobacterium* was positively correlated with only valeric acid and isovaleric acid (Table 2). The main SCFAs – acetic acid, propionic acid and butyric acid – were correlated positively, likewise, the main BCFAs – iso-butyric acid, 2-methyl butyric acid and iso-valeric acid – were correlated with each other (Table 2).

**Table 2.** Correlation of short chain fatty acids and faecal bacteria in children at the beginning of the study; baseline (*n* = 140). Bold values indicate statistically significant correlations, after Bonferroni correction.

		Total bacteria	*Lactobacillus*	*Bifidobacterium*	Acetate	Propionate	Butyrate	Valerate	Isobutyrate	2-methyl butyrate	Isovalerate	Lactate
Total bacteria	r	1	0.233	0.300	−0.019	0.127	0.230	0.296	0.546	0.380	0.509	−0.406
	*p*	**0**	0.006	**0.000**	0.831	0.147	**0.008**	**0.001**	**0.000**	**0.000**	**0.000**	**0.000**
*Lacto* *bacillus*	r	0.233	1	0.478	0.003	−0.034	0.206	0.409	0.232	0.066	0.192	0.051
	*p*	0.006	**0**	**0.000**	0.975	0.702	0.019	**0.000**	**0.009**	0.463	0.033	0.587
*Bifidobacterium*	r	0.300	0.478	1	0.031	−0.110	0.081	0.269	0.142	0.140	0.254	0.073
	*p*	**0.000**	**0.000**	**0**	0.728	0.209	0.362	**0.003**	0.115	0.120	**0.004**	0.438
Acetate	r	−0.019	0.003	0.031	1	0.658	0.587	0.104	−0.027	−0.151	−0.040	−0.022
	*p*	0.831	0.975	0.728	**0**	**0.000**	**0.000**	0.251	0.764	0.094	0.657	0.813
Propionate	r	0.127	−0.034	−0.110	0.658	1	0.430	0.089	−0.099	−0.257	−0.056	−0.151
	*p*	0.147	0.702	0.209	**0.000**	**0**	**0.000**	0.323	0.268	**0.004**	0.538	0.104
Butyrate	r	0.230	0.206	0.081	0.587	0.430	1	0.411	0.195	0.072	0.290	−0.034
	*p*	**0.008**	0.019	0.362	**0.000**	**0.000**	**0**	**0.000**	0.029	0.428	**0.001**	0.720
Valerate	r	0.296	0.409	0.269	0.104	0.089	0.411	1	0.607	0.459	0.514	−0.228
	*p*	**0.001**	**0.000**	**0.003**	0.251	0.323	**0.000**	**0**	**0.000**	**0.000**	**0.000**	0.017
Isobutyrate	r	0.546	0.232	0.142	−0.027	−0.099	0.195	0.607	1	0.787	0.787	−0.420
	*p*	**0.000**	**0.009**	0.115	0.764	0.268	0.029	**0.000**	**0**	**0.000**	**0.000**	**0.000**
2-methyl butyrate	r	0.380	0.066	0.140	−0.151	−0.257	0.072	0.459	0.787	1	0.716	−0.216
	*p*	**0.000**	0.463	0.120	0.094	**0.004**	0.428	**0.000**	**0.000**	**0**	**0.000**	0.024
Isovalerate	r	0.509	0.192	0.254	−0.040	−0.056	0.290	0.514	0.787	0.716	1	−0.288
	*p*	**0.000**	0.033	**0.004**	0.657	0.538	**0.001**	**0.000**	**0.000**	**0.000**	**0**	**0.002**
Lactate	r	−0.406	0.051	0.073	−0.022	−0.151	−0.034	−0.228	−0.420	−0.216	−0.288	1
	*p*	**0.000**	0.587	0.438	0.813	0.104	0.720	0.017	**0.000**	0.024	**0.002**	**0**

Spearman rank correlation was performed to study the relation between all variables such as faecal bacteria and short chain fatty acids. r (rho) values indicate correlations and bold p-values indicate significance at 95% CI.

After the nine-month intervention with *L. paracasei* Lpc-37, acetic, propionic and butyric acid were found to correlate with each other, likewise isobutyric, 2-methylbutyric and isovaleric acid were found to correlate to each other (Table S3). After intervention with *B. lactis* HN019, in particular total bacteria and lactobacilli were found to correlate to SCFAs and BCFAs. Also here, BCFA isobutyric, 2-methylbutyric and isovaleric acid were found to correlate to each other (Table S4). In the placebo group, total bacterial counts were found to correlate to BCFAs and BCFAs were found to correlate to each other (Table S5).

## Discussion

In Western adults, the intestinal microbiota contributes an estimated 10% of daily energy requirements from nutrition; mainly in the form of SCFAs;[16] this depends on the dietary fibre intake. *In vitro* studies indicate that toddler microbiota may ferment fibre faster than an adult microbiota but produces less SCFA.[17] Besides being an energy source, SCFA also provide other benefits to the host such as regulating microbiota composition and activity, influencing colonic physiology and signalling (e.g. satiety).[18] The current study aimed to investigate the link between faecal SCFAs, nutritional status, diarrhoea risk and faecal *Bifidobacterium* and *Lactobacillus* levels in 2–5-year-old children in India.

We observed no difference in SCFAs, BCFAs or *Bifidobacterium* and *Lactobacillus* levels between the genders. Although this would be expected, to our knowledge, it is the first time that such a comparison has been made for otherwise healthy children.

When comparing faecal SCFA or *Bifidobacterium* and *Lactobacillus* levels between individuals of different nutritional status, we observed an increased level of the *Lactobacillus* genus in the undernourished sub-population. This is likely rather the effect than the cause of the malnutrition. Earlier reports are not consistent on this point; poor nutritional status has been associated with lower levels of faecal lactobacilli [19] or no difference in number of colonised subjects.[20] While the previous studies relied on culturing or used fluorescent *in situ* hybridisation (FISH), the present study relied on quantitative real time PCR; it is possible that this has contributed to the difference. However, it is likely to relate to the study population; as we reported earlier the faecal *Lactobacillus* levels were exceptionally high in this population.[8] We observed that in the *B. lactis* HN019 group levels of faecal acetate increased over time. Although *B. lactis* HN019 can produce acetate from hexose fermentation, this is not likely to be the source of the increased levels as a similar increase was observed in the placebo group. In the group that received *L. paracasei* Lpc-37, isobutyrate, 2-methyl butyrate and isovalerate increased over time. These metabolites are formed by amino acid fermentation and *L. paracasei* is not known to perform such metabolism. These increases were regardless of whether the children experienced diarrhoea during the study or not. It is not certain if this is related to maturation of the children or because of other reasons. In the group that consumed *B. lactis* HN019, propionic acid changed during the study, levels increased in the group not experiencing diarrhoea while it was reduced in the group suffering diarrhoea. There is little known about the effect of maturation of children on faecal levels of SCFAs; the findings here suggest that it would be relevant to follow cohorts of healthy children over time to gain more insight into this matter.

In the placebo group, no differences in SCFAs were observed between those children who experienced diarrhoea and those who did not, for any of the time points. In the probiotic groups, however, post-intervention, those children that had experienced diarrhoea were found to have higher levels of faecal acetate and propionate (*B. lactis* HN019) and higher levels of isobutyrate and isovalerate (*L. paracasei* Lpc-37). It should be noted that at the time of sampling the children were not experiencing diarrhoea and the increased levels of some SCFAs are thus not due to impaired absorption, but may indicate a differential influence of the probiotics have on children who experienced diarrhoea compared to those who did not. Although the differences in SCFAs were significant, they were in general small; the clinical relevance is therefore uncertain.

The significant, mainly positive, correlations of total bacteria, lactobacilli and bifidobacteria with several of the SCFAs are may be not surprising as the SCFAs are produced by the intestinal microbiota. It is, however, interesting that the SCFAs that correlate with bifidobacteria and lactobacilli are not produced by these genera; thus indicating cross feeding and a wider influence on the intestinal microbiota activity.[21] The observation that SCFAs positively correlate with each other, and BCFAs likewise, might be expected. Less expected is maybe that SCFAs were not found to be correlated negatively with BCFAs. One would expect that higher production of SCFAs would be accompanied by a lower production of BCFAs, and *vice versa*, SCFAs being produced mainly from a saccharolytic fermentation and BCFAs from amino acid fermentation, in particular in the absence of fibre as an alternative to saccharolytic fermentation.

After the intervention with the probiotics, correlations were observed between the used strains and total bacterial counts and *Bifidobacterium* levels; for *B. lactis* HN019 and *L. paracasei* Lpc-37, respectively, counts of these organisms were found to increase after the intervention.[8] In the placebo group, the counts of these organisms did not change over time [8] and were only correlated to iso-valeric acid, lactic acid and propionic acid. It is uncertain to what extent these correlations have contributed to the reduced incidence of fever and diarrhoea during the rainy season that was observed.[8]

In conclusion, the current paper highlights the importance of measuring faecal SCFAs in children; a population that has received limited attention as far as microbiota composition and activity is concerned. The present study found that *Lactobacillus* levels are increased in severely underweight children; as malnourishment continues to be a challenge in many parts of the world, the meaning of this deserves further attention. Furthermore, we observed that supplementation with *L. paracasei* Lpc-37 or *B. lactis* HN019 induced a differential response in faecal BCFAs and SCFAs, respectively, which was not observed in the placebo group. This differential metabolic response may, in part, explain the effect the probiotics had on diarrhoea and fever risk.

## Supplementary Material

Supplementary material
