# Supplementary material for: Effect of probiotic supplementation on total lactobacilli, bifidobacteria and short chain fatty acids in 2–5-year-old children
Source: Microb Ecol Health Dis. 2017 Mar 10;28(1):1298340. doi: 10.1080/16512235.2017.1298340 (PMC5443088; doi:10.1080/16512235.2017.1298340)
Supplement: Supplementary material [file zmeh_a_1298340_sm2481.pdf]

Supplemental material

Table S1: Faecal bacteria and SCFAs in children (2 -5 years) by gender at baseline.

| | **Male (n=67)** | **Female (N=73)** | **Total (n=140)** |
|---|---|---|---|
| **Total bacteria** | 10.24±0.46 | 10.35±0.31 | 10.29±0.39 |
| ***Lactobacillus*** | 9.75±1.40 | 9.94±1.14 | 9.85±1.27 |
| ***Bifidobacterium*** | 8.94±0.95 | 9.10±0.52 | 9.03±0.76 |
| **Acetic acid** | 73.44±27.31 | 75.01±23.53 | 74.24±25.36 |
| **Propionic acid** | 37.78±22.46 | 38.6±18.36 | 38.2±20.39 |
| **Butyric acid** | 24.76±15.41 | 22.98±11.98 | 23.84±13.71 |
| **Valeric acid** | 4.35±4.23 | 3.18±3.20 | 3.74±3.76 |
| **Isobutyric acid** | 1.46±1.68 | 1.29±1.09 | 1.38±1.41 |
| **2-methylbutyric acid** | 0.95±1.13 | 0.71±0.67 | 0.83±0.92 |
| **Isovaleric acid** | 1.51±1.51 | 1.33±0.93 | 1.42±1.24 |
| **Lactic acid** | 16.85±32.01 | 10.18±18.82 | 13.20±25.75 |

[1]Bacterial counts expressed as log10/gram of faecal sample.

[2]SCFA Values are mean given in mmol/kg wet weight faeces

Table S2: Lactobacilli, bifidobacteria and SCFA in relation to nutritional status of children (2-5 years) at baseline.

| | Weight for Age | | | |
|---|---|---|---|---|
| | Normal N=89 | Moderately Underweight N=36 | Severely Underweight N=15 | P-value |
| **Total bacteria** | 10.28±0.38 | 10.32±0.42 | 10.42±0.34 | 0.19 |
| ***Lactobacillus*** | 9.79±1.30 | 9.93±1.22 | 10.57±0.79 | 0.03 |
| ***Bifidobacterium*** | 9.06±0.68 | 8.96±0.70 | 9.15±0.43 | 0.62 |
| **Acetic acid** | 75.96±24.5 | 71.7±27.0 | 70.51±23.89 | 0.43 |
| **Propionic acid** | 38.24±18.91 | 38.3±23.2 | 39.20±17.24 | 0.85 |
| **Butyric acid** | 24.54±13.88 | 22.36±13.26 | 20.80±8.13 | 0.38 |
| **Valeric acid** | 3.55±3.98 | 4.07±3.46 | 4.15±3.48 | 0.58 |
| **Isobutyric acid** | 1.25±1.49 | 1.57±1.24 | 1.55±1.19 | 0.46 |
| **2-methylbutyric acid** | 0.75±1.01 | 0.95±0.77 | 0.81±0.65 | 0.83 |
| **Isovaleric acid** | 1.30±1.35 | 1.57±1.07 | 1.48±0.91 | 0.62 |
| **Lactic acid** | 13.17±25.99 | 13.42±25.91 | 14.86±22.49 | 0.81 |

[1]Bacterial counts expressed as log10/gram of fecal sample.

[2]SCFA Values are mean given in mmol/kg wet weight faeces

WAZ: weight for age Z score. WAZ >-2 SD was considered Normal; WAZ ≤-2 to -3 was considered Moderately Underweight; WAZ <-3 SD was considered Severely Underweight

**Table S3 After *L. paracasei* Lpc-37 supplementation.** Correlation of short chain fatty acids and faecal bacteria in children at the beginning of the study; baseline. (N=51). Bold values indicate statistically significant correlations, after Bonferroni correction.

| | | tot bac | Lactobacillus | L. paracasei | Bifidobacterium | B. lactis | Aceticacid | Propionicacid | Butyric acid | Valeric acid | Isobutyricacid | 2methylbutyricacid | Isovalericacid | Lacticacid |
|---|---|---|---|---|---|---|---|---|---|---|---|---|---|---|
| Total bacteria | r | 1.000 | -.023 | -.023 | .277 | .445 | -.115 | -.010 | .173 | .358 | .569 | .384 | .586 | -.116 |
| | p | **0** | .873 | .877 | .049 | **.001** | .435 | .947 | .238 | .016 | **.000** | .009 | **.000** | .465 |
| Lactobacillus | r | -.023 | 1.000 | -.006 | .282 | .213 | .219 | .214 | .251 | .307 | -.037 | -.102 | .036 | -.014 |
| | p | .873 | **0** | .970 | .045 | .142 | .136 | .144 | .085 | .040 | .807 | .503 | .815 | .929 |
| L. paracasei | r | -.023 | -.006 | 1.000 | .330 | .120 | -.066 | -.143 | -.096 | .196 | .002 | -.059 | -.020 | .053 |
| | p | .877 | .970 | **0** | .022 | .423 | .668 | .349 | .532 | .213 | .991 | .712 | .899 | .742 |
| Bifidobacterium | r | .277 | .282 | .330 | 1.000 | .449 | .015 | -.015 | .066 | .082 | .118 | .103 | .216 | -.120 |
| | p | .049 | .045 | .022 | **0** | **.001** | .917 | .918 | .655 | .592 | .434 | .501 | .154 | .448 |
| B. lactis | r | .445 | .213 | .120 | .449 | 1.000 | -.087 | -.010 | .156 | .156 | .206 | .185 | .186 | .043 |
| | p | .001 | .142 | .423 | .001 | **0** | .562 | .948 | .295 | .313 | .176 | .229 | .226 | .790 |
| Aceticacid | r | -.115 | .219 | -.066 | .015 | -.087 | 1.000 | .858 | .738 | .305 | -.232 | -.392 | -.235 | -.167 |
| | p | .435 | .136 | .668 | .917 | .562 | **0** | **.000** | **.000** | .039 | .116 | .007 | .116 | .284 |
| Propionicacid | r | -.010 | .214 | -.143 | -.015 | -.010 | .858 | 1.000 | .696 | .224 | -.357 | -.547 | -.432 | -.046 |
| | p | .947 | .144 | .349 | .918 | .948 | **.000** | **0** | **.000** | .135 | .014 | **.000** | **.003** | .768 |
| Butyricacid | r | .173 | .251 | -.096 | .066 | .156 | .738 | .696 | 1.000 | .342 | .004 | -.181 | .001 | -.140 |
| | p | .238 | .085 | .532 | .655 | .295 | **.000** | **.000** | **0** | .020 | .981 | .229 | .993 | .371 |
| Valericacid | r | .358 | .307 | .196 | .082 | .156 | .305 | .224 | .342 | 1.000 | .471 | .240 | .290 | -.045 |
| | p | .016 | .040 | .213 | .592 | .313 | .039 | .135 | .020 | **0** | **.001** | .117 | .053 | .781 |
| Isobutyricacid | r | .569 | -.037 | .002 | .118 | .206 | -.232 | -.357 | .004 | .471 | 1.000 | .933 | .826 | -.284 |
| | p | **.000** | .807 | .991 | .434 | .176 | .116 | .014 | .981 | **.001** | **0** | **.000** | **.000** | .072 |
| 2methylbutyricacid | r | .384 | -.102 | -.059 | .103 | .185 | -.392 | -.547 | -.181 | .240 | .933 | 1.000 | .811 | -.057 |
| | p | .009 | .503 | .712 | .501 | .229 | .007 | **.000** | .229 | .117 | **.000** | **0** | **.000** | .728 |
| Isovalericacid | r | .586 | .036 | -.020 | .216 | .186 | -.235 | -.432 | .001 | .290 | .826 | .811 | 1.000 | -.299 |
| | p | **.000** | .815 | .899 | .154 | .226 | .116 | **.003** | .993 | .053 | **.000** | **.000** | **0** | .061 |

| | | | | | | | | | | | | | | |
|---|---|---|---|---|---|---|---|---|---|---|---|---|---|---|
| Lacticacid | r | -.116 | -.014 | .053 | -.120 | .043 | -.167 | -.046 | -.140 | -.045 | -.284 | -.057 | -.299 | 1.000 |
| | p | .465 | .929 | .742 | .448 | .790 | .284 | .768 | .371 | .781 | .072 | .728 | .061 | **0** |

**Table S4 After *B. lactis* HN019 supplementation.** Correlation of short chain fatty acids and faecal bacteria in children at the beginning of the study; baseline. (N=44). Bold values indicate statistically significant correlations, after Bonferroni correction.

| | | tot bac | Lactobacillus | L. paracasei | Bifidobacterium | B. lactis | Aceticacid | Propionicacid | Butyricacid | Valericacid | Isobutyricacid | 2methylbutyricacid | Isovalericacid | Lacticacid |
|---|---|---|---|---|---|---|---|---|---|---|---|---|---|---|
| Total bacteria | r | 1.000 | .295 | .467 | .487 | .320 | -.348 | -.131 | .050 | .657 | .847 | .782 | .884 | -.257 |
| | p | **0** | .052 | .021 | **.001** | .034 | **.028** | .421 | .761 | **.000** | **.000** | **.000** | **.000** | .148 |
| Lactobacillus | r | .295 | 1.000 | 0.000 | .497 | .180 | -.279 | -.210 | .005 | .343 | .445 | .501 | .500 | .050 |
| | p | .052 | **0** | 1.000 | **.001** | .243 | .081 | .194 | .978 | .032 | **.005** | **.002** | **.001** | .782 |
| L. paracasei | r | .467 | 0.000 | 1.000 | .348 | .151 | -.074 | .191 | -.027 | .588 | .311 | -.085 | .395 | -.414 |
| | p | .021 | 1.000 | **0** | .096 | .480 | .758 | .420 | .910 | .008 | .196 | .747 | .094 | .098 |
| Bifidobacterium | r | .487 | .497 | .348 | 1.000 | .239 | -.096 | -.141 | .243 | .331 | .444 | .299 | .446 | -.172 |
| | p | **.001** | **.001** | .096 | **0** | .119 | .554 | .386 | .130 | .040 | .005 | .077 | **.004** | .339 |
| B. lactis | r | .320 | .180 | .151 | .239 | 1.000 | -.118 | -.082 | .137 | .287 | .290 | .153 | .269 | -.323 |
| | p | .034 | .243 | .480 | .119 | **0** | .468 | .617 | .400 | .077 | .073 | .373 | .097 | .067 |
| Aceticacid | r | -.348 | -.279 | -.074 | -.096 | -.118 | 1.000 | .708 | .307 | -.100 | -.261 | -.502 | -.344 | .174 |
| | p | .028 | .081 | .758 | .554 | .468 | **0** | **.000** | .051 | .539 | .103 | **.002** | .030 | .325 |
| Propionicacid | r | -.131 | -.210 | .191 | -.141 | -.082 | .708 | 1.000 | .345 | .072 | -.163 | -.369 | -.127 | .076 |
| | p | .421 | .194 | .420 | .386 | .617 | **.000** | **0** | .027 | .660 | .315 | .024 | .434 | .671 |
| Butyricacid | r | .050 | .005 | -.027 | .243 | .137 | .307 | .345 | 1.000 | .118 | -.053 | -.229 | .090 | -.055 |
| | p | .761 | .978 | .910 | .130 | .400 | .051 | .027 | **0** | .469 | .747 | .172 | .581 | .759 |
| Valericacid | r | .657 | .343 | .588 | .331 | .287 | -.100 | .072 | .118 | 1.000 | .698 | .511 | .645 | -.348 |
| | p | **.000** | .032 | .008 | .040 | .077 | .539 | .660 | .469 | **0** | **.000** | **.001** | **.000** | .048 |
| Isobutyricacid | r | .847 | .445 | .311 | .444 | .290 | -.261 | -.163 | -.053 | .698 | 1.000 | .922 | .897 | -.35 |
| | p | **.000** | .005 | .196 | .005 | .073 | .103 | .315 | .747 | **.000** | **0** | **.000** | **.000** | .044 |

| | | | | | | | | | | | | | | |
|---|---|---|---|---|---|---|---|---|---|---|---|---|---|---|
| 2methylbutyricacid | r | .782 | .501 | -.086 | .299 | .153 | -.502 | -.369 | -.229 | .511 | .922 | 1.000 | .883 | -.162 |
| | p | **.000** | **.002** | .747 | .077 | .373 | **.002** | .024 | .172 | **.001** | **.000** | **0** | **.000** | .393 |
| Isovalericacid | r | .884 | .500 | .395 | .446 | .269 | -.344 | -.127 | .090 | .645 | .897 | .883** | 1.000 | -.200 |
| | p | **.000** | **.001** | .094 | **.004** | .097 | .030 | .434 | .581 | **.000** | **.000** | **.000** | **0** | .265 |
| Lacticacid | r | -.257 | .050 | -.414 | -.172 | -.323 | .174 | .076 | **-.055** | **-.348** | **-.353** | **-.162** | **-.200** | **1.000** |
| | p | .148 | .782 | .098 | .339 | .067 | .325 | .671 | .759 | .048 | .044 | .393 | .265 | **0** |

**Table S5. After placebo supplementation.** Correlation of short chain fatty acids and faecal bacteria in children at the beginning of the study; baseline. (N=44). Bold values indicate statistically significant correlations, after Bonferroni correction.

| | | tot bac | Lactobacillus | L. paracasei | Bifidobacterium | B. lactis | Aceticacid | Propionicacid | Butyricacid | Valeric acid | Isobutyricacid | 2methylbutyricacid | Isovalericacid | Lacticacid |
|---|---|---|---|---|---|---|---|---|---|---|---|---|---|---|
| Total bacteria | r | 1.000 | .131 | .314 | .500 | .112 | -.367 | -.265 | -.111 | .662 | .743 | .753 | .700 | -.613 |
| | p | **0** | .410 | .127 | **.001** | .480 | .016 | .086 | .480 | **.000** | **.000** | **.000** | **.000** | **.000** |
| Lactobacillus | r | .131 | 1.000 | .159 | .387 | .147 | -.325 | -.290 | -.010 | .235 | .155 | .233 | .040 | -.128 |
| | p | .410 | **0** | .447 | .011 | .360 | .038 | .066 | .950 | .145 | .346 | .153 | .802 | .438 |
| L. paracasei | r | .314 | .159 | 1.000 | .152 | -.069 | -.284 | -.268 | .041 | .052 | .324 | .376 | .503 | -.440 |
| | p | .127 | .447 | **0** | .467 | .742 | .178 | .206 | .850 | .809 | .131 | .070 | .012 | .031 |
| Bifidobacterium | r | .500 | .387 | .152 | 1.000 | .147 | -.321 | -.406 | -.138 | .230 | .181 | .208 | .223 | -.444 |
| | p | **.001** | .011 | .467 | **0** | .360 | .036 | **.007** | .378 | .143 | .258 | .191 | .151 | **.004** |
| B. lactis | r | .112 | .147 | -.069 | .147 | 1.000 | -.180 | -.382 | -.198 | .174 | .257 | .189 | .134 | .024 |
| | p | .480 | .360 | .742 | .360 | **0** | .261 | .014 | .216 | .282 | .115 | .249 | .404 | .887 |
| Aceticacid | r | -.367 | -.325 | -.284 | -.321 | -.180 | 1.000 | .761 | .416 | -.017 | -.242 | -.447 | -.236 | .245 |
| | p | .016 | .038 | .178 | .036 | .261 | **0** | **.000** | .006 | .914 | .128 | **.003** | .127 | .127 |
| Propionicacid | r | -.265 | -.290 | -.268 | -.406 | **-.382** | .761 | 1.000 | .521 | -.017 | -.293 | -.421 | -.223 | .210 |
| | p | .086 | .066 | .206 | .007 | **.014** | **.000** | **0** | **.000** | .914 | .063 | .006 | .151 | .194 |
| Butyricacid | r | -.111 | -.010 | .041 | -.138 | -.198 | .416 | .521 | 1.000 | .100 | .029 | -.045 | -.062 | -.027 |
| | p | .480 | .950 | .850 | .378 | .216 | .006 | **.000** | **0** | .528 | .858 | .781 | .694 | .870 |

| | | | | | | | | | | | | | | |
|---|---|---|---|---|---|---|---|---|---|---|---|---|---|---|
| Valericacid | r | .662 | .235 | .052 | .230 | .174 | -.017 | -.017 | .100 | 1.000 | .771 | .705 | .556 | -.232 |
| | p | **.000** | .145 | .809 | .143 | .282 | .914 | .914 | .528 | **0** | **.000** | **.000** | **.000** | .155 |
| Isobutyricacid | r | .743 | .155 | .324 | .181 | .257 | -.242 | -.293 | .029 | .771 | 1.000 | .948 | .773 | -.403 |
| | p | **.000** | .346 | .131 | .258 | .115 | .128 | .063 | .858 | **.000** | **0** | **.000** | **.000** | .012 |
| 2methylbutyricacid | r | .753 | .233 | .376 | .208 | .189 | -.447 | -.421 | -.045 | .705 | .948 | 1.000 | .817 | -.431 |
| | p | **.000** | .153 | .070 | .191 | .249 | **.003** | .006 | .781 | **.000** | **.000** | **0** | **.000** | .007 |
| Isovalericacid | r | .700 | .040 | .503 | .223 | .134 | -.236 | -.223 | -.062 | .556 | .773 | .817 | 1.000 | -.442 |
| | p | **.000** | .802 | .012 | .151 | .404 | .127 | .151 | .694 | **.000** | **.000** | **.000** | **0** | **.004** |
| Lacticacid | r | -.613 | -.128 | -.440 | -.444 | .024 | .245 | .210 | -.027 | -.232 | -.403 | -.431 | -.442 | 1.000 |
| | p | **.000** | .438 | .031 | **.004** | .887 | .127 | .194 | .870 | .155 | .012 | .007 | **.004** | **0** |

Spearman rank correlation was performed to study the relation between all variables such as fecal bacteria and short chain fatty acids.
r (rho) indicates correlations and p indicates significance at 95% CI.

Significances are indicated in bold. *Correlation is significant at the 0.05 level (2-tailed). **Correlation is significant at the 0.01 level (2-tailed).
